# To know or not to know? Mentalization as protection from somatic complaints

**DOI:** 10.1371/journal.pone.0215308

**Published:** 2019-05-02

**Authors:** Sergi Ballespí, Jaume Vives, Naida Alonso, Carla Sharp, María Salvadora Ramírez, Peter Fonagy, Neus Barrantes-Vidal

**Affiliations:** 1 Department of Clinical and Health Psychology, Universitat Autònoma de Barcelona, Barcelona, Catalonia, Spain; 2 Department of Psychobiology and Methodology of Health Sciences, Universitat Autònoma de Barcelona, Barcelona, Catalonia, Spain; 3 Department of Psychology, University of Houston, Houston, United States of America; 4 Department of Psychology & Lang Sciences, University College London, London, United Kingdom; 5 Department of Mental Health, Fundació Sanitària Sant Pere Claver, Barcelona, Catalonia, Spain; 6 Centre for Biomedical Research Network on Mental Health (CIBERSAM), Instituto de Salud Carlos III, Madrid, Spain; University of Groningen, NETHERLANDS

## Abstract

Somatization processes are usually associated with a lack of insight or with emotional unawareness, especially in adolescents where the ability for self-reflection is beginning to mature. However, the extent to which different levels of insight explain variations in somatization remains understudied. This study aimed to evaluate whether high-level emotional awareness (comprehension) but not low-level awareness (only attention) is needed to psychologically cope with suffering, thus leading to lower somatization. Specific predictions were: 1) High attention along with High comprehension will be associated with significantly lower frequency of somatic complaints than other combinations (Low attention and Low comprehension, or High attention but Low comprehension); 2) In absence of comprehension, no attention will be more optimal than attention only, because only-attention might work as an amplificatory of suffering without the possibility of processing it. Self-reports of meta-cognitive processes, somatization, and control variables were obtained from 264 adolescents from a non-clinical population (54.5% female; aged 12–18, *M* = 14.7, SD = 1.7). In line with expectations, results revealed significant differences in the effects of insight positions on somatization: *Attention+Comprehension* (*M* = 4.9, SE = 0.9) < *Nothing* (*M* = 7.1, SE = 0.3) < *Only attention* (*M* = 8.9, SE = 0.7). Compared to *Nothing*, *Attention+comprehension* was associated with significantly reduced somatic complaints (*B* = -2.2, p = 0.03, 95% CI -4,1 to 0.2). However, *Only attention* was associated with increased somatic complaints compared to the other two conditions (*B* = 1.8, p = 0.03, 95% CI 0.2 to 3.4; *B* = 4, CI 95% 1.6–6.3, p = 0.001, respectively). This highlights the role of higher-order awareness (i.e., comprehension or clarity) in the processing of suffering and stresses its value in the adaptive coping of emotional distress.

## Introduction

The awareness of one’s own mental states—also referred as insight, metacognition, or self-awareness—is present as a therapeutic aim in most psychological treatments [1–5]. Insight is broadly defined as the ability to reflect upon and manage one’s emotions, and to utilize the information provided by these emotions adaptively [6]. Insight has been associated with therapeutic adherence [7,8], and it constitutes a core active ingredient of most psychodynamic interventions, including, for instance, Mentalization Based Treatment (MBT) [9]. This highlights the importance of insight for mental health and spurs interest in how it works, i.e., how it contributes to healthy psychological functioning.

The association between insight and emotional regulation [10–12] suggests that insight and self-awareness might have an effect in the processing of suffering. Insight (i.e., the capacity to be aware of one’s own mental states and to understand them) might contribute to really 'digest' suffering or, in other words, to truly process it, unlike other forms of dealing with emotional distress.

There are other ways in which humans deal with suffering (e.g., distraction, repression, aggression, somatization), but they are usually associated with psychopathological symptoms [13–15] and therefore considered less healthy. In fact, it can be assumed that emotional suffering is usually expressed through dysfunctional forms20—maladjusted behavior (e.g., aggressions, self-aggression, substance abuse), somatic symptoms—precisely when it cannot be mentalized [16–20]. This supports the idea that emotional insight prevents maladaptive forms of coping with suffering because it helps to ‘digest’ suffering, i.e., it helps to face and understand emotions instead of ignoring, avoiding, or expelling them.

Research on somatization supports this idea. Since the pioneering studies with alexithymia [21,22], evidence for an inverse association between emotional awareness and psychosomatic processes has been provided [23]. Problems of naming one’s own emotions [24,25] and recognizing others’ mental states [26–28] have been found in several psychosomatic processes such as somatization [29,30], chronic pain [31,32] or conversion disorder [33,34], amongst others [35–37]. This association is consistently evidence-based for different stages (children, adolescents, adults) [38,39], and across different approaches to the cognitive process involved. However, the lack of consensus in referring to this higher order cognition (e.g., emotional awareness, facial perception, mood consciousness, theory of mind, alexithymia, self-knowledge, meta-cognition, emotional intelligence, amongst others) impedes precisely identifying what aspects or types of “emotional awareness” are considered in different studies.

It is possible that different degrees of awareness have different effects on the ‘metabolism’ of suffering. Subic-Wrana and colleagues [27], for example, found that general levels of emotional awareness are intact in many patients with somatoform problems, but they suffer more specific impairments in awareness, such as the inability to link different emotional and bodily states. Therefore, emotion recognition does not seem to be impaired in patients with somatizing disorders, but they are not able to *interpret* physical sensations as negative emotional states, thus evidencing a lack of emotional clarity or deeper comprehension. This study points out the importance of distinguishing different dimensions of a complex process such as emotional awareness and reveals that somatization might be more associated with a problem of clarity or meaning rather than simple emotion recognition.

However, as in many studies in the field of somatic complaints (e.g., [26–28,30]), Subic-Wrana and colleagues analyzed emotion recognition regarding others’ emotions, probably because emotional awareness is more easily measured regarding others’ emotions than regarding ones’ own. This might explain why there is a gap in the literature regarding the awareness of one’s own emotional states.

Despite the interdependence among self and others’ dimensions of emotional awareness [40], which allows inference based on others’ recognition, we assume that emotional awareness of one’s own suffering might be more associated with somatization. Still, to our knowledge, besides the relatively lower attention towards emotional awareness of one’s own states, there are no studies analyzing how *different dimensions* of emotional awareness referred to one’s own emotional states (meta-mood cognition) are associated with somatic complaints. The aim of this study was to fill in this gap.

The awareness of one’s own mental states is a complex function involving several dimensions and processes [41,42], and it cannot be assumed that the simple awareness of the existence of an emotional state is equivalent to its clarity or comprehension, which we consider to be what helps to cope with suffering through ‘emotional digestion’. Moreover, it is possible that a ‘superficial’ form of awareness (i.e., simple attention to emotional cues but without clarity or comprehension) may not help to cope with suffering, or may even act by amplifying it—as occurs when excessive attention to anxiety contributes to increase anxiety—while ‘deeper’ forms of awareness, including higher processing (i.e., comprehension), may help to process suffering, thus reducing it. This is something suggested in the classic studies of Mayer and Salovey in the construction of their meta-mood cognition scale [6,12].

Mayer and Salovey [43–45] suggested that the process of emotional metacognition is composed by a sequence of 3 mechanisms: attention, clarity, and repair. Although all 3 mechanisms contribute to meta-cognition [6], authors distinguish between “the individual’s willingness to attend to feelings” (i.e., monitoring moods, the degree of attention devoted to feelings), their capacity to “understand one’s mood”, that is, “to experience these feelings clearly” (i.e., discrimination, perception, deeper comprehension), and “their beliefs about terminating negative mood states or prolonging positive ones” (repair or regulation).

In light of the above, the aim of the present study was to test whether different degrees of emotional awareness differentially affect the tendency to somatize. We assumed that what has a beneficial effect on the processing of suffering is not simple emotional awareness but higher-order insight, that is, emotional clarity or comprehension. In terms of Mayer and Salovey’s model, we considered that simple attention to emotional states constitutes a form of superficial awareness that does not necessarily help to process suffering. By contrast, when attention is accompanied by emotional comprehension, a different level of insight is possible.

Salovey and Mayer developed a scale based on their model of meta-mood experience. The Trait Meta-Mood Scale (TMMS) [6] is one of the few instruments to assess awareness of one’s own emotional states, as the measurement of emotional awareness is more widely based on the recognition of others’ emotions (e.g., [46–52]). The TMMS is also one of the most widely used measures to this extent, probably the one with more evidence for its validity and reliability (e.g., [53–58]) and, to our knowledge, the TMMS is the only scale allowing to distinguish different sub-processes of meta-mood cognition (attention, clarity, repair) in the same instrument. Therefore, to operationalize our assumptions, we used a compound measure of meta-cognition [6, 53] based on the dimensions of the TMMS.

On this base, we compared the frequency of somatization associated with three different conditions: 1) High attention and High comprehension of emotional states operationalized what we consider the most complete option of emotional awareness, i.e., not simple attention but further processing of emotions. We predicted this position might be associated with the lowest frequency of somatic complaints. 2) The opposite position (Low attention and Low comprehension) was operationalized as the option with lowest emotional awareness, so it was expected to be associated with a higher frequency of somatization than the first condition (“insight”). 3) Finally, we considered that High attention to emotional states, accompanied by Low capacity to comprehend them is probably the worst position against suffering, because it focuses on emotional reactions without the possibility to really deal with them. We consider that this case places one in a position of emotional awareness permeability (High attention), but without the possibility of defending oneself from suffering through further processing (Low comprehension; so, we assume no psychological digestion). This is expected to amplify instead of reduce suffering, so we predicted that it might be associated with an even higher frequency of somatization.

To simplify reference to these conditions, briefer structures will be sometimes used from here, like *Attention+Comprehension* or *Insight condition* instead of High attention and High comprehension; *Only attention* instead of High attention but Low comprehension, or *Nothing* instead of Low attention and Low comprehension. These briefer labels merely aim to improve clarity by avoiding the repetition of complex combinations (e.g., High attention but Low comprehension).

**Fig 1:**
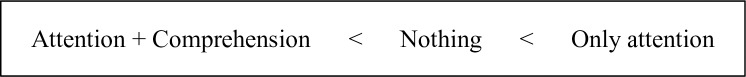
Prediction of frequency of somatic complaints among the three conditions.

Overall, we expected that the best psychological option to deal with suffering, thus reducing the tendency to somatize, is high attention and high comprehension. In the absence of high emotional processing (comprehension), we expected that a defensive position of no attention and no comprehension (“out of sight, out of mind”), is better than attention without further comprehension, because this means a defenseless permeability (attention but no clarity) that amplifies emotional indigestion, and therefore the probability of somatizing. Fig 1 expresses these predictions visually.

The fourth possible condition (Low attention + High comprehension) is conceptually odd from a sequence perspective and implies a sort of an unconscious or automatic insight, so it was not considered in our predictions. This option was analyzed from an exploratory point of view.

In conducting our study, we were especially interested in the relation among insight and somatization problems as they manifest in adolescents. Somatic symptoms are considered altered expressions of emotional distress when people are unable to express emotional states [15,59,60]. That’s why somatization is frequent in childhood, when cognition, language, and especially mentalizing capacities are still in development [61]. However, given the intense transition that adolescence constitutes, somatic complaints are even more common in this stage [62,63]. Besides, adolescence is known as an important developmental period for self-reflective processes to emerge [64,65]. These facts along with the predictive value of somatization for further psychopathology [66,67], and the interest to identify developmental psychopathology as early as possible [68], imply that adolescence may be a particularly important developmental period to observe the effects of insight on somatization processes because: 1) somatization is especially frequent in adolescence, probably because insight capacities are not fully developed, though 2) these capacities are more developed than in childhood, and 3) early intervention is still possible within this developmental period in order to prevent more severe adult problems.

In evaluating our study hypotheses, we also considered the effects of neuroticism and internalizing psychopathology as these are recognized as main contributors to somatic complaints [69–72]. Therefore, all the analyses controlled for the effects of these variables. Moreover, sex, age, and socio-economic status (SES) are associated with psychopathology and also with insight. Due to gender socialization, sex is a variable closely related both to somatization [73,74] and insight [75,76]. Specifically, girls are expected to show better capacities to realize, name and think about emotions [77,78], especially in adolescence, because they mature before boys. Additionally, insight is a higher order function that becomes more complex with age [79,80], so older adolescents (participants were aged 12 to 18 years old) were expected to show better insight capacities than younger ones. Finally, SES is a general risk factor for psychopathology [81–84] and it has also been specifically associated both with somatization [85] and with emotional insight [86,87]. Consequently, to reduce other sources of variation that might affect the focus of the study, sex, age and SES were also controlled for in all the analyses.

## Materials and methods

### Ethics statement

The study meets ethical standards according to Declaration of Helsinki and it has been approved by the Ethics Committee of the Universitat Autònoma de Barcelona (CEEAH num. 2603, Spain). All families provided written informed consent. In all cases, informed consent was provided from both adolescents and their parents or guardians.

### Participants

The sample consisted of 264 adolescents (144 girls, 54.5%) aged 12 to 18 years old (M = 14.7, SD = 1.7) from the general population that voluntarily agreed to participate in the study. From those, 42.4% were aged 12–13 years old; 33.0% were aged 14–15; and 24.6% were aged 16–18. Among girls, 35.4% girls were 12–13 years old; 36.8% girls were 14–15 years old; 27.8% girls were 16–18 years old. Among the boys, 50.8% boys were 12–13 years old; 28.3% were 14–15 years old; 20.8% were 16–18 years old. This sample was recruited through schools in the context of a broader project about psychopathology, personality and coping strategies in adolescence. Somatic complaints are frequent in adolescence [88], so this warranted variability in a sample from non-clinical population. The basic inclusion criterion in the current study was to be between 12 to 18 years of age. The exclusion criterion was presence of severe mental illness such as psychosis, autism spectrum disorder, or intellectual disability. Recruitment was carried out through the schools to simplify logistics. Ten schools of similar characteristics (not rural, similar size, similar families’ SES, similar educational orientation and methodologies, geographically close to each other) were invited to participate in the project according to their proximity to the research center. The possibility to count on a wide eligible sample considering the risk of low rates of participation was also a motivation to select 10 schools (i.e., in Catalonia the participation of families in school matters is usually low, ranging from 10% to 20%). Five of these schools agreed to collaborate, and n = 266 families signed the informed consent to participate in the study. The principal reasons from families who refused were low interest in the project, being too busy, refusal to give data about mental health or, in the case of some immigrant families, the inability to understand at least one of the two languages of the questionnaires (i.e., Spanish or Catalan). It was possible to obtain full data from adolescents in the 99% of cases (n = 264). Approximately 71% came from families with middle socio-economic level (11.6% low; 17.7% high) and approximately 87% were Caucasian (White-European), 9% Arabic, 2% Asian, and 2% Latino.

### Measures

#### *Trait Meta-Mood Scale* (TMMS-24)

This is one of the few instruments to assess awareness of one’s own emotional states, probably the one with the best evidence for its psychometric properties and, to our knowledge, the only one assessing different sub-processes of meta-mood experience in the same scale. This widely used scale assesses attention, comprehension (emotional clarity) and beliefs about one’s own mental states [6,53]. We used the short version of 24 items because: a) it suited our necessity of relatively brief instruments for a broad project in which all informants had to fill out several scales, and b) this version counts on more extended use than the original one, with adaptations to several cultures (e.g., [57,58,89,90]) and with more evidence of good psychometric properties in the Spanish adaptation (e.g., [53,89–92]). It consists of 24 items scored from 1 to 5 according to the degree of agreement. In addition to a total score, the TMMS provides scores for the 3 dimensions of meta-cognition defined through factor analysis [6] according to the model of Mayer and Salovey (attention, comprehension and repair) [43,44]. The scores in the first two dimensions (attention and comprehension) are those used here to consider the contribution of different degrees of awareness on somatic complaints. The scale of attention includes items such as *‘I pay a lot of attention to how I feel*’. The scale of clarity or comprehension includes items like *‘I almost always know exactly how I am feeling*’. The third process (regulation or repair) assesses beliefs about emotional regulation (e.g., *‘Although I am sometimes sad*, *I have an optimistic outlook’*). This third scale was not involved in current hypotheses. The Spanish version of the TMMS-24 shows good internal consistency (Cronbach’s α from .86 to .90) and adequate test-retest reliability (ICC from .60 to .83) [53]. Excellent internal consistency in the current study (α = .90) supports the reliability of this scale.

#### *Somatic Symptoms Questionnaire* (SOM)

This instrument measures frequency of the most common somatic complaints in childhood and adolescence (headache, dizziness, stomach pain, fatigue and muscle pain) in the three months prior to the evaluation. It can be answered by caregiver [93] and self-reported by children and adolescents [62]. Evidence supports good psychometric properties in Spanish samples for both versions [93,94]. The self-report used in the present study showed adequate reliability for the evaluation of adolescents (Cronbach’s α = .80) [62] (α = .70 in the present study).

#### *Big Five Inventory* (BFI)

This is a 44-item inventory that measures five predominant dimensions of personality (extraversion, convenience, consciousness, neuroticism and opening) [95] through 5-point Likert scales ranged from “*strongly disagree”* to “*strongly agree”*. Both the original and the Spanish version show good psychometric properties [96,97]. The neuroticism scale was used in the current study as a control variable. The internal consistency of this scale was good in the Spanish version (Cronbach’s α = 0.80) [96] (α = .77 in the present study).

#### *Beck Depression Inventory 2* (BDI)

This is a widely used inventory based on 21 self-evaluative items, each with three symptom-choices reflecting the respondent’s experience over the course of a week [98]. The Spanish adaptation [99] shows good psychometric properties (e.g., Cronbach’s α = .87) and the internal consistency in the current sample was excellent (Cronbach’s α = .90).

#### *Multidimensional Anxiety Scale for Children* (MASC) [100]

This is a self-evaluative 39 item questionnaire that measures anxiety symptoms in children and adolescents using a 4-point Likert scale ranging from 0 (not at all true about me) to 3 (it is often true about me). Its psychometric properties have been tested in large samples from several countries and are good for the evaluation of adolescents [101]. It shows excellent internal consistency (Cronbach’s α = .90) [100] (α = .88 in the present study).

#### *Social Anxiety Scale for Adolescents* (SAS-A) [102]

Given the prevalence of social anxiety (SA) in adolescence, this measure was also included beyond general anxiety as a control variable. SAS-A consists of 18 self-statements referred to SA (e.g., “I worry about what others think of me”) and 4 filler items. All of them are rated on a 5-point Likert scale according to how much the item “is true for you”, ranging from 1 (not at all) to 5 (all the time). The psychometric properties of the Spanish adaptation are good [103], with internal consistency ranging from α = .76 and α = .91 (α = .90 in the present study).

### Procedure

The study meets ethical standards according to Declaration of Helsinki and the revision of the Ethics Committee of the Universitat Autònoma de Barcelona (CEEAH num.2603, Spain). All families provided written informed consent for the different parts of the broad project called “Personality, psychopathology and coping strategies in adolescence”. In the case of the current study, families were informed about objectives, relevance, and implications through a letter widespread by the school and were also invited to a meeting to solve any doubts regarding the study. In all cases, informed consent was provided from both adolescents and their parents or guardians. After obtaining the informed consent, data were recruited in the schools to simplify logistics. The participants received the questionnaires in closed envelopes with their identity encrypted with alphanumeric codes and were given a deadline to return them. Missing values and out-of-range values were detected in order to contact the participants to rectify them. The data collection took approximately five weeks in every school, occurring between January and June of 2013.

### Statistical analysis

The degree of insight was operationalized as a combination of 2 dimensions of this capacity: the attention to emotional states, and clarity (comprehension) [6]. These variables were dichotomized before combining them to make predictions. The main reason for this transformation is based on reliability. We think that a subjective measure of a subjective process (meta-cognition) that involves this process in the assessment, is not so refined as to provide a reliable quantitative measure. The measurement of emotional awareness is mostly operationalized in the reading of others’ mental states, probably because this allows to present people with standardized materials (pictures, cards, films, animations). This is the case of well-established measures such as the Movie for the Assessment of Social Cognition [46], the Reading the Mind in the Eyes [48] or The Frith-Happé-Animation task (FHAT) [47]. However, it is difficult to operationalize one’s own subjectivity, and therefore it also is the cognition about one’s own subjectivity, which leads to the necessity of using self-reports. This introduces an abnormality: one’s own meta-cognition is assessed using one’s own meta-cognition, so nor the process neither the object of assessment is objectifiable for an external observer.

This is why the assessment of social cognition is mostly based on “reading” others. We do not want to renounce to assess subjectivity, because we think that it is the metabolism of one’s own suffering what is mostly associated with somatization. However, we do not rely on subjectivity providing a refined quantitative measure of one’s own meta-cognition. Accordingly, we transformed the quantitative self-reported measures of attention and comprehension into dichotomous variables based on distinguishing only High / Low. Beyond the apparent loss of information, we benefit from a simpler, more trustworthy measure.

The 75^th^ percentile was used as a cut-off for dichotomization. This allowed us to clearly identify and group those participants with a good comprehension and attention, while keeping enough participants in each group to perform the data analyses. Since the interest was focused on the protective value of emotional insight, if high level of insight processes (i.e., above the 75^th^ percentile) did not play a role, it could be deduced that neither will low levels.

According to the scores of attention and comprehension (scores above the 75^th^ percentile were considered high in both dimensions), 4 insight conditions could be distinguished. Those with High attention and High comprehension were considered those with the best combination to deal with suffering according to the hypothesis (Fig 1). By contrast, those with Low attention and Low comprehension were those with the least awareness. According to the hypothesis, the non-mentalization of suffering is expected to be associated with more somatization. However, the least optimal mental position regarding somatic complaints was expected to be that with High attention to emotions but Low capacity to process them. Thus, High attention but Low comprehension was expected to be associated with the highest frequency of somatic complaints. This option leads to a position of “defenseless permeability” that allows “seeing but not digesting”, so people in this position are expected to become flooded with “unprocessed” emotion. Finally, the group with Low attention and High comprehension was not considered in the hypothesis because it refers to a sort of automatic emotional insight (comprehension without attention).

Linear regressions were performed to test the effect of emotional insight on somatic complaints. All regression models included as potential confounding variables the main contributors to somatic complaints, i.e., neuroticism (operationalized as the score in the neuroticism subscale of the BFI), and internalizing problems (operationalized as the scores derived of BDI, MASC and SAS-A), as well as sex, age, and SES (operationalized through the Hollingshead’s index) [104].

Regression backward model selection was conducted, using IBM SPSS Statistics v20.0 package (IBM Corp, 2011) [105] to fit each model. The results of the association between MZ categories and frequency of somatic complaints are presented as linear regression coefficients (B) for quantitative responses, reporting 95% confidence intervals (95% CI), and P-values (P). The polychotomous variable of emotional insight position was entered the regression model as a dummy variable taking Low attention + Low comprehension as the reference category.

## Results

Table 1 shows descriptive statistics and correlations of the variables involved in the analyses. Sex distribution consisted of 57% males in the group with High attention and High comprehension, 46% in the group with Low attention and Low comprehension, 15% in the group with High attention but Low comprehension, and 70% in the group with Low attention but High comprehension. Among the 264 participants, only 23 participants show the most advantageous position (High attention along with High comprehension). The most frequent MZ position was Low attention and Low comprehension (n = 163), while ‘partial positions’ were present in 41 (High attention but Low comprehension) and 37 (Low attention but High comprehension) participants, respectively. As expected, somatization is positively correlated with attention but negatively with comprehension.

**Table 1:** Descriptives and correlations.

	Descriptives *M* (SD)				Correlations								
	Att+Comp n = 23	Nothing n = 163	Only att n = 41	Only comp n = 37	1	2	3	4	5	6	7	8	9
1. Somatization	4.4 (4.2)	6.9 (4.4)	10.6 (6.2)	5.0 (4.3)	-								
2. MC—Total	100.2 (7.7)	66.1 (11.6)	81.1 (12.6)	87.6 (8.8)	-0.15*	-							
3. MC—Attention	32.3 (3.2)	19.8 (5.1)	32.4 (2.8)	20.9 (4.9)	0.17**	0.64**	-						
4. MC—Comprehension	35.7 (3.1)	21.8 (5.1)	21.7 (5.6)	34.9 (3.0)	-0.25**	0.79**	0.21**	-					
5. Neuroticism	19.8 (6.1)	23.0 (4.8)	26.6 (4.9)	19.3 (4.9)	0.31**	-0.15*	0.28**	-0.31**	-				
6. Depression	5.7 (7.0)	8.8 (7.3)	15.2 (8.9)	4.5 (5.3)	0.44**	-0.18**	0.27**	-0.35**	0.47**	-			
7. Anxiety	43.4 (12.2)	40.7 (14.1)	51.3 (15.4)	39.7 (16)	0.34**	0.01	0.33**	-0.21**	0.39**	0.43**	-		
8. Social anxiety	42.1 (13.3)	45.3 (12.0)	51.7 (14.4)	41.8 (15)	0.22**	-0.11	0.22**	-0.28**	0.35**	0.40**	0.68**	-	
9. Age	169.6 (18.8)	175.2 (22.3)	184.8 (15.8)	165.3 (15.5)	0.15*	-0.06	0.21**	-0.14*	0.13*	0.28**	-0.01	0.08	-
10. SES	2.7 (1.3)	2.6 (1.2)	3.0 (1.3)	2.6 (1.3)	0.09	0.04	0.12	-0.06	-0.02	0.09	0.17*	0.14*	0.09

MC = Meta-cognition. *Att+Comp* = High attention + High comprehension; *Nothing* = Low attention + Low comprehension; *Only att* = High attention + Low comprehension; *Only comp* = Low attention + High comprehension.

*P < 0.05

**P < 0.005.

**Fig 2:**
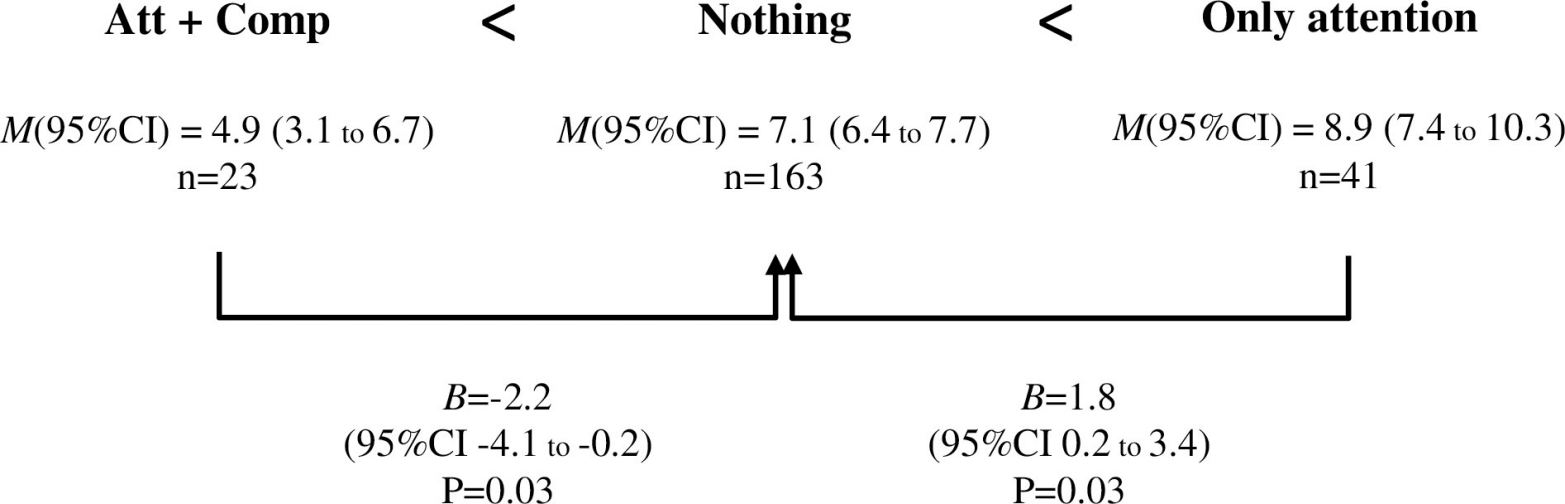
Effect sizes of each insight category on somatic complaints taking Low attention + Low comprehension as the reference category. Mean frequencies of somatic complaints within insight positions and effect sizes of each insight category on somatic complaints taking Low attention + Low comprehension as the reference category, and controlling for age, sex, SES, neuroticism, anxiety and depression. Notes: High attention and High comprehension (Att + Comp); Low attention and Low comprehension (Nothing); High attention but Low comprehension (Only attention). Adjusted mean (*M*); Linear regression coefficients (*B*), 95% mean confidence intervals (95% CI), and p values (P).

Fig 2 shows that mean frequency of somatic complaints differs among insight positions according to hypotheses. Thus, High attention and High comprehension (*Att+Comp*) shows a lower frequency of somatization (Adjusted *M* = 4.9) compared to Low attention and Low comprehension (*Nothing*) (Adjusted *M* = 7.1). Therefore, “insight” compared to “blind” position reduces somatic complains (*B* = -2.2, CI 95% -4.1 to -0.2, p = 0.03). *Only attention*, with an Adjusted *M* = 8.9, is the worst situation, since it increases somatic complaints compared to *Nothing* (*B* = 1.8, CI 95% 0.2 to 3.4, p = 0.03) and also to *Attention+Comprehension* (*B* = 4, CI 95% 1.6–6.3, p = 0.001). Given that it was not considered in the hypotheses, the figure does not include the fourth insight position (Low attention, High comprehension), which in terms of somatic complaints (Adjusted *M* = 6.1; 95%CI: 4.6–7.5) is placed between *Attention+Comprehension* and *Nothing*

## Discussion

The aim of this study was to analyze the association between different types of emotional awareness and somatization in adolescence–a developmental period characterized by the growth of somatization problems as well as an increased capacity for psychological insight. Specifically, the frequency of somatizations in cases of: a) attention along with comprehension, b) attention only but without comprehension, and c) low general awareness (no attention + no comprehension) were compared.

In line with our predictions (Fig 1) the combination of high attention and high comprehension was associated with the lowest frequency of somatic complaints compared to the other options. This supports the idea that comprehension beyond ‘simple attention’ is needed to reduce somatization. This is reinforced by the fact that the option based on only high attention (with no high comprehension) is associated with the highest frequency of somatic complaints. This suggests that attention followed by emotional clarity or comprehension might diminish somatic complaints, while attention alone increases them. In light of these findings, attention without comprehension is not only not protective against somatization, but actually exacerbates it.

A possible explanation is that attention without comprehension might lead to a sort of emotional indigestion, or a state of emotional overloading due to a lack of emotional processing. This might explain why the absence of higher order processing (comprehension) following simple emotional awareness (attention) is associated with the highest level of somatization. By contrast, attention followed by comprehension might help to process suffering, thus making “unnecessary” other ways of draining it, such as somatization.

The findings reported here can be interpreted in the broader context of mentalization-based approaches to psychopathology [18,41,106–112]. Mentalizing is a multi-dimensional construct that refers to an imaginative activity by which we reflect on our own minds and the minds of others [9, 10,42,113]. The dimension of mentalization referring to one’s own mental states constitutes a meta-cognitive process [114–117], and while it denotes a broader set of processes than assessed through the TMMS-24 [6,12,43], the attention and comprehension of one’s own emotional states are part of this set. That’s why the Mentalization paradigm provides a helpful conceptual framework for interpreting our findings. Besides, it enables translational conclusions to be drawn given the fact that mentalizing is the treatment target of Mentalization-based Therapy.

Our results, interpreted in the context of mentalization-based theory [42,114,117] suggest that mentalizing (or reflection on) of emotional states prevents somatization because comprehension plays a role in the emotional metabolism, while the non-mentalization of emotional states fosters somatization, especially when emotions are detected but not understood. This is consistent with the idea that when people cannot “digest” what they see, not paying attention to that content can be a better option. This is consistent with the popular advice “out of sight, out of mind”, as well as the use of distracting CBT techniques to reduce suffering. However, since current results highlight the internal process of “comprehending” as an important step towards “true insight”, implications for mental health should be considered.

The pending question of “to know or not to know?”, or whether self-knowledge benefits mental health, against the usually recommended option of “out of sight, out of mind”, should be answered in light of intermediate processes and individual differences. Considering the “full emotional awareness” or insight as a complex process involving different mechanisms (for instance, attention as a low-level consciousness, followed by comprehension as a reflective meta-level involving meaning) [6,43], individual differences regarding this capacity might also be more complex than a simple issue of degree. That is, if people differ not only in their general level of mentalization but in their style to address emotional states (true insight, mind-blindness, overwhelmed …), then this implies that the best treatment might not be necessarily equal for everybody, but it might depend on the capacities of the patient. This idea has already been highlighted in the literature [118]. In the current case, for instance, it leads one to wonder if all patients with high somatization, who are assumed to show low mentalizing capacity, might equally benefit from a mentalization-based treatment. This is especially important in adolescence, when somatic complaints are highly frequent [62,63] but mentalizing capacities are still in development and therefore may vary substantially [77–80,86]. This state of flux likely introduces even more individual differences than in adulthood making more critical the election of treatment [119].

In light of current results, if comprehension is a good mechanism to deal with suffering and therefore reduces dysfunctional phenomena such as somatization, then people able to comprehend might benefit from treatments based on insight. However, assuming that even in lack of high comprehension, a treatment focused on mentalizing could be elected, there is the additional question of whether treatment approaches should be designed equally for the two groups with high somatization, independently of the presence of high attention. This leads to the interesting question of whether it is possible to mentalize without attention. Three considerations should be presented regarding this issue.

First, from a sequential perspective of the achievement of insight [6], assuming that ‘comprehension’ was built upon a pre-existing ‘attention’ stage, then the condition of low general insight (low attention and low comprehension) should be the worst-case scenario in terms of mentalizing, because it lacks both necessary steps in the way to insight (attention and then comprehension) compared to the position that includes attention (even without comprehension). However, our findings support that attention without comprehension is worse than nothing in terms of somatization. In fact, the condition with only attention predicts significantly more somatization than all the other conditions. So, paradoxically, adolescents in the low general awareness condition (low attention and low comprehension) could be more prone to benefit from a mentalization-focused treatment (because they are not overwhelmed by emotion) than those with only attention, prone to become flooded by exaggerated emotional states. This should be qualified from a developmental perspective. Since this is a sample of adolescents and they experience greater emotionality than adults (i.e., they become more easily overwhelmed by emotions) this possibility should be also analyzed in adults to test the influence of developmental issues. For instance, it should be examined if also adults with “high attention but low comprehension” become so “overwhelmed” as adolescents might do, thus producing more somatic complaints than other insight positions.

Second, it should be considered that the tendency to view impaired mentalizing as a function of increased emotional arousal is not necessarily a cognitive pattern or a stable trait, but it can be associated with context-dependent variations. This might explain why problems of emotional dysregulation can be successfully treated with MBT (Mentalization Based Treatment) [111,120]: because there are some preserved mentalizing abilities beyond situational mentalizing problems. This has been suggested for the case of Borderline Personality Disorder [109], and it is also the case of somatoform patients with impairment in embodied MZ [121].

Embodied mentalizing is the capacity to see the body as the seat of emotions, and the capacity to reflect on one’s own bodily experiences and sensations [121]. Evidence supports that the impairments of embodied mentalizing in somatoform patients are specifically related to interpersonal situations and experiences that involve high arousal or stress [121], but not to all situations. Since the current study is based in on one way of operationalizing mentalizing processes through a focus on insight, future studies may benefit of assessing context-dependent variations of mentalizing capacities across time. Once more, this is especially interesting in adolescence for the developmental reasons already mentioned (i.e., more emotional instability and more variable mentalizing capacities). Thus, it is attractive to speculate that: a) emotional instability is higher than in adulthood, so there is “more to mentalize”, b) excessive emotional arousal might situationally impair base mentalizing capacities more frequently (so, there is “more emotionality to mentalize” but lower capacity to do it, because mentalizing is still developing in adolescence and it probably becomes more frequently impaired by excessive emotion) and, therefore, c) beyond the developmental variability in base mentalizing capacities, higher situational variation should be considered. The assessment of these developmental and context-dependent variations may shed new light in future studies.

Third, the question of whether it is possible to mentalize without attention leads to considering the fourth position (high comprehension without high attention). It is interesting to point out that this kind of awareness, involving high comprehension, is characteristic of 1/3 more participants (i.e., n = 36) than *Insight* (n = 23), suggesting that high comprehension is not only possible without high attention, but it is even more common than with high attention, and it reflects some sort of automatic or implicit mentalizing ability [42]. The presence of such an ability to this extent in our sample: 1) supports the idea of a dual process [122], and 2) suggests that it is an extensive phenomenon, leading to the question of whether people tend to mentalize more implicitly than explicitly, and 3) whether “implicit mentalizing” is better or worse than explicit emotional insight.

The mean of somatic complaints of *Comprehension without attention* falls between the insight position (*Attention+Comprehension*) and low general awareness (*No attention neither comprehension*). This suggests that high comprehension alone (“implicit mentalizing”) might be better than high attention alone, which in fact is associated with the worst cognitive position regarding somatization. This reinforces the idea that higher order awareness (comprehension) is needed to metabolize suffering and reduce somatic complaints, even in absence of high attention. This interesting result deserves further attention in future studies. Given the proximity of the means, the position of *“Implicit mentalizing”* also suggests that implicit or automatic emotional awareness is not better than explicit insight in this context of somatic complaints.

The point discussed above is reasonable from the perspective that “true” emotional insight, understood as high-level emotional processing, might involve a high level of both processes here assessed (i.e., high attention and high comprehension). Nevertheless, expertise is usually associated with the automaticity of processes in all of life’s dimensions [123]. Accordingly, it might be attractive to speculate that the sophistication of fully explicit mentalizing through practice and time means the automaticity of some processes, which then become implicit because they require less conscious cognitive resources (i.e., they require lower attention), thus releasing cognitive capacity for higher-order emotional processing.

Current results do not support this. Since the full position (High attention and High comprehension) predicts the lowest level of somatic complaints, it is odd to assume that this position is only a stage towards an apparently less efficient position in terms of somatization (*Implicit mentalizing*). In fact, since this is a sample of adolescents and high-order mentalizing capacities are achieved later in life [80], it could be hypothesized that “true insight” is infrequent here because it is a final stage of mentalizing development still not achieved in adolescence. The predominance of positions without comprehension might also explain why children and adolescents tend to somatize more than adults. To shed light on these developmental issues, this study should be replicated with adults. Since mentalizing is expected to be more developed in adults, and adults tend to somatize less than adolescents, it could be hypothesized that adults will show less prevalence in the “permeability” condition (attention without comprehension, the worst position) and a higher frequency of the “insight” condition.

Along with the necessity of replication, current limitations should be considered. The main one is the difficulty of measuring awareness or insight of one’s own emotional states. There are few measures that assess meta-cognition of one’s own mental states, and all of them are based on self-reports; and of course, to inform about one’s own meta-cognition, meta-cognition is needed. This is probably why most studies of somatization operationalize emotional awareness through the recognition of others’ emotional states instead of the self. Therefore, future research should improve methods to assess emotional awareness, self-insight or self-mentalizing, thus allowing replication of current findings with more refined measures, or even making possible an experimental design using controlled lab situations. The existence of more accurate measures might also make possible addressing new questions, such as whether high attention contributes to difficulties in comprehension (clouding judgment), or whether comprehension is possible without attention (automatic mentalizing). Moreover, since the lack of precision attributed to a self-reported measure led us to carry out analyses with dichotomous variables (high/low), a more refined “objective” measure of mentalizing referred to one’s own mental states might make possible the analysis of continuous variables. A lab situation, despite decreasing ecological validity (because the emotional states needed to be meta-recognized might have to be induced), may improve reliability. Finally, regarding measurement of higher order cognition, the stability of mentalizing abilities should also be assessed, along with situational variations of these capacities. This might allow analysis of context-dependent variations in somatic complaints. As a second limitation, since the sample used is self-selected and based on adolescents, generalization to general population is not possible. In fact, all current results must be considered in light of the developmental issues mentioned elsewhere. Additionally, voluntary participants recruited through schools for unpaid studies are usually healthier than the general population. Thus, those with more risk of somatization and those with lower mentalizing skills tend to self-exclude from participation. Therefore, an epidemiological study with a more representative sample of different stages of development is necessary to verify current findings and address new questions, such as whether people tend to use more explicit or implicit mentalizing, or which one is better to deal with suffering. A fourth limitation is the lack of control of other sources of variability. Although anxiety, depression and neuroticism have been included in the current study as control variables, further variables should be considered in future studies. For instance, chronic stress, which has been defined as a precipitating factor for somatoform disorders [121], or social support, which might be associated to emotional socialization [124].

Consequently, future studies with refined measures and representative samples should capture in greater depth the meta-cognitive abilities involved in emotional processing and the ‘metabolism’ of suffering, as well as its relationship with somatoform processes. In this sense, the assessment of mentalizing using Experience Sampling Methodology (ESM) [125] might help to improve the ecology, stability and context-dependence of meta-cognitive measures, despite the fact that it does not solve the lack of standardization of mentalized stimuli (i.e., one’s own subjectivity). Therefore, future research should also address this pending question, which involves operationalizing (through experimental induction) or objectifying—if this is possible—individual subjectivity.

## Conclusions

Despite limitations, this study is the first to our knowledge to analyze the separate contribution of two levels of awareness of one’s own emotional states (attention vs. comprehension or clarity) to the frequency of somatic complaints. Current results confirm predictions and are consistent with previous findings: mentalizing appears to be protective against somatization, and the inability to mentalize is associated with a higher frequency of somatic complaints. Furthermore, our findings support that different degrees of insight explain variations in the frequency of somatic complaints, and highlight that 1) comprehension is needed to achieve a level of insight associated with reduced somatization, probably because it helps to process suffering through psychological skills, and 2) low-level of emotional awareness (simple attention but without comprehension) is not only insufficient to prevent somatization, but it can even be the least optimal option, since it probably works as an amplification system and it is associated with the highest frequency of somatic complaints. These results have been obtained with a sample of adolescents from the general population, which means higher tendency to somatize and more developmental variability in mentalizing capacities than in other stages. Knowing that, age and sex were controlled for in all analyses, results were explained in key of development, and need for replication with new samples (e.g., adults, clinical) has been pointed out. Beyond these considerations, this is the first study to highlight the role of comprehension beyond simple attention in the processing of suffering, and to stress the value of emotional understanding (higher-order awareness) in adaptive coping with emotional distress.

## Supporting information

S1 File 20190423 Data MZ-somat.sav. This is the file from which all data analyses were performed.
(SAV)
